# “The lockdown of physical co-operation touches the heart of adult education”: A Delphi study on immediate and expected effects of COVID-19

**DOI:** 10.1007/s11159-020-09871-w

**Published:** 2020-11-06

**Authors:** Bernd Käpplinger, Nina Lichte

**Affiliations:** Justus-Liebig-Universität Giessen, Fachbereich 03 Sozial- und Kulturwissenschaften, Institut für Erziehungswissenschaft, Professur für Weiterbildung, Gießen, Germany

**Keywords:** Delphi study, adult education, adult learning, COVID-19, digital education, financing, future of adult education and learning

## Abstract

This article is based on the first wave of an ongoing worldwide Delphi study which is currently analysing the immediate and expected effects of the COVID-19 pandemic on adult education and adult learning. While the methodology of Delphi studies varies a lot, in a nutshell, the core idea of a Delphi study is that it explores the future of a particular field in a collaborative way. The authors contacted more than 50 international experts in the field of adult education for a qualitative online survey between April and May 2020, asking them to provide information, observations, expectations and advice. While the findings show many cross-national similarities, there are also many differences. Clearly, adult educators are still trying to understand the implications of the crisis, which they perceive as unprecedented.

## Résumé

« Le verrouillage de la coopération physique touche l’éducation des adultes en plein cœur » : une étude Delphi sur les effets immédiats et attendus de la COVID-19 – Cet article s’appuie sur le premier volet d’une étude Delphi menée actuellement à l’échelle mondiale et qui analyse les effets immédiats et attendus de la pandémie de COVID-19 sur l’éducation et l’apprentissage des adultes. Bien que les méthodes Delphi varient considérablement, on peut résumer l’idée essentielle de ces études à l’exploration collaborative d’un domaine particulier. Les auteurs ont contacté plus de 50 experts internationaux dans le domaine de l’éducation des adultes dans le cadre d’une enquête en ligne réalisée entre avril et mai 2020 pour les prier de leur fournir des informations et des conseils, et de leur communiquer leurs observations et attentes. Bien que les résultats présentent de nombreuses similarités entre les pays, on constate également de nombreuses différences. Clairement, les éducateurs d’adultes s’efforcent encore de comprendre les conséquences d’une crise à leurs yeux sans précédent.

## Introduction

Adult education was and continues to be severely affected by the public measures and directives issued by governments all over the world to curb the spread of the COVID-19 pandemic. For example, many adult education organisations and learning centres have had to close down for weeks or even months during lockdowns and other restrictions of access. While the effect on learners has been a frequent subject of media coverage, commentators have also called for financial aid, especially to protect the most vulnerable groups like precariously employed freelance instructors or organisations which provide longer educational retreats from bankruptcy. There is growing concern that many adult education organisations will struggle to survive as the pandemic continues to make restrictive measures necessary. On the other hand, there are also voices which expect or demand *forced digitalisation* (e.g. Witt 2020).^1^ A number of national studies already conducted on the adult education segment of enterprise-provided continuing education indicate that enterprises now prioritise investment in digital distance education, while decreasing investment in other forms of training (e.g. Flake et al. 2020). Another growing concern, voiced for example by the Portuguese Association of Women Studies (APEM), are the negative effects of the crisis on the situation of women, e.g. in terms of interrupted projects and contracts, the challenges of teleworking, etc. (APEM 2020).

The medium- and long-term effects of this manifold crisis are far from clear, but the scope of expectations ranges from being gloomy to hopeful. To get a clearer picture of the effects of this global crisis, it is important to adopt a perspective that goes beyond local or national circumstances and outlooks. Despite the primarily national character of counter-strategies aiming to curb the spread of the virus, the interconnectedness of countries and regions will certainly not be broken by COVID-19, even if backlashes are likely to happen and nationalism or even discrimination might increase.

Against this background, this article presents the findings of the first cycle of a Delphi study we (the authors of this article) conducted to understand the impact of the COVID-19 crisis on adult education around the world. Below, we begin with an explanation of our specific Delphi study approach, which we used for this research. It is important to note that the Delphi study method varies a lot. Next, we present our main findings in relation to present and expected effects of the COVID-19 crisis. We conclude our article with a comparison of findings from different countries and an outline of the next steps of this ongoing research project.

## Methods

### The Delphi technique

As indicated above, this article is based on the first qualitative results of a Delphi study. Delphi studies are traditionally used in adult education research (e.g. Rossman and Bunning 1978). The core idea of a Delphi study is to explore *future* themes – in our context, issues at stake in adult education – in a collaborative way. The goal and procedures of a well-known earlier comparative Delphi study on the future of adult education (conducted in 14 countries between 1993 and 1995) were described as follows:

The Euro-Delphi project is attractive and promising because of the fact that it draws practitioners and policy actors from the outset into a process of reflexive discussion and common decision. Therefore, the term “respondent” does not adequately describe this type of involvement (Leirman 1996, p. 137).

In general, a Delphi study approach (Carr-Chellmann and Kroth 2018, Crawford and Wright 2016, Steinmüller 1997) is a research method that involves asking experts for their views on a particular aspect of their field of expertise. In a systematic, multi-stage progression, this method is repeated over several rounds. The purpose is sometimes to arrive at a kind of distilled experts’ consensus which can be used for predicting trends, probable developments and likely innovations. This aspect of forecasting led to this method being named after the ancient Greek Oracle of Delphi.

Nonetheless, there is no clear definition of the Delphi method. Rather, multiple variants and adaptations exist. For example (quotes are all cited by Häder 2002, p. 20):

- The Delphi technique is a questionnaire method for organizing and sharing opinion through feedback (Bardecki 1984, p. 281).
- An alternative means of accessing expert opinion and evaluating incomplete information is the Delphi technique, a systematic procedure for soliciting the advice of a number of experts, and forging a consensus from that advice (Richey et al. 1985, p. 136).
- [The Delphi technique is] an accepted method of achieving consensus among experts (Duffield 1993, p. 227).

The method we apply in our own Delphi study is similar to that described by Bardecki, while Duffield’s approach of striving for consensus was not our goal in this international and multicultural study.

### Preparation and implementation of the first wave of our own Delphi study

For our own study, we decided to consult only leading scholars in adult education, excluding policymakers and practitioners, who are usually included in other Delphi studies. Our goal was (and still is, since this is an ongoing study) to share opinions and expertise internationally and to promote mutual feedback. While it was possible that consensus might be achieved, it is not our pre-defined goal to reach it. On the contrary, we rather expected diversity and the divergence of opinions, which is entirely acceptable since countries and people are differently affected by COVID-19 and the measures to counter the virus vary from country to country. The potential goal of achieving consensus would bear the risk of hiding a hegemonic approach, which is not our intention in this study. Nonetheless, we did receive a number of responses which indicated a certain level of unease about how the results of this study might be used by us. Moreover, the value of forecasts even when they are made by experts is questionable in light of misleading and wrong predictions in the past, as demonstrated by the following examples:

- “There is not the slightest sign that we can ever develop nuclear energy” (Albert Einstein in 1932; quoted in Navasky 1996)
- “We don’t like their sound, and guitar music is on the way out (Decca Records about the Beatles in” 1962).^2^
- “Children just aren’t interested in witches and wizards any more” (Publishing executive told J.K. Rowling in 1996).^3^

Is it possible that another quote from 2020 and in relation to COVID-19 will be added to this list in a few years’ time? Nonetheless, we have to be bold to envisage the future in order to make decisions on present challenges and alternatives. It might be much more dangerous not to think about the future than to fail desperately sometimes when forecasting events.

### Selection of participant experts

We selected our participants among the experts in the field of research on adult education and adult learning by inviting people mainly from academic societies or associations in adult education and adult learning research. We asked them for their qualitative assessment of the immediate and expected effects for adult education of the public measures against COVID-19. In a next step, the results of this first (qualitative) survey will be transferred into a new questionnaire, which will then again be presented to experts from different countries and continents (e.g. some contributors to this year’s cancelled international “Adult Education in Global Times” conference, AEGT2020).^4^ As mentioned earlier, unlike the intention and execution of many other Delphi studies (e.g. Blieck et al. 2018), it is not our goal to achieve consensus within this survey. As already stated, our intention is rather to encourage dialogue and to become more aware of the differences and the different situations worldwide and even to imagine different future scenarios (Häder 2002; Gutschow and Jörgens 2019).

### Designing our questionnaire

We designed our questionnaire for the first, qualitative round of our Delphi study and conducted a small pre-test with two people which resulted in changes to the wording of the questions. The first wave of the survey started on 21 April 2020, and we received the last response on 23 May 2020. The survey was done online, in English and (in one case) Spanish, via the tool and software LimeSurvey. The survey contained these seven questions:

- *What are the three main effects on adult education and adult learning of the measures taken against COVID-19 that you presently observe?*
- *What are the three main effects on adult education and adult learning of the measures taken against COVID-19 that you expect in future?*
- *Are there any links or publications (position papers, statements, etc.) in relation to the present situation of adult education or adult learning in your country or region which you consider as being important?*
- The next three questions were on age, gender and nationality
- *Do you have any other comments or recommendations for us? For example, what shall we consider when constructing the questions for phases two and three of this Delphi study?*

We invited 54 (29 female, 25 male) experts active worldwide in adult education and adult learning research to participate. It was only possible to participate after having been personally invited.^5^ The data we present below refer to 27 fully or partly completed questionnaires, representing 19 different nationalities. Respondents’ average age was 54 years, which is rather high and should therefore be taken into account, though it does of course reflect the fact that we contacted mainly established scholars and experts. The gender ratio was balanced (14 female, 13 male), and all continents are represented (see Table 1). By far the largest share of respondents came from Europe, which is overrepresented, while the response rates from other regions were much lower, with the response rate from Asia being the lowest:

**Table 1:** Sample and response rates of the Delphi study

World regions	Invited sample	Responses
Africa	4	2 (50%)
Asia	8	2 (25%)
Australia/New Zealand	4	2 (50%)
Europe	27	16 (59%)
North America	9	4 (44%)
South America	2	1 (50%)
Gender		
Female	29	14 (48%)
Male	25	13 (52%)
Average age of respondents	unknown	54

The second and third waves of our Delphi survey are scheduled for the summer/autumn 2020. The underrepresentation of some world regions within the first wave of our survey is problematic and additional measures will be taken to achieve a better worldwide representation also beyond Europe. While the results of the first Delphi survey presented here will inform the second and third waves, we intend to widen the sample, because of the flaws in terms of global representation as already mentioned. The design of the next steps is not completely defined yet.^6^ The review of this article and comments by reviewers will also inform the next steps. The study as a whole thus has a number of participatory elements, which is in line with our intention to encourage a dialogue among experts worldwide.

## Findings

### Immediate effects of COVID-19

Our expert respondents observed a significant effect of the COVID-19 crisis on adult education and adult learning. A core statement they made was: “The lockdown of physical co-operation touches the heart of adult education” (ID 22).^7^ Most teaching or guidance activities in physical presence had to be cancelled, which led to “[d]iminished face-to-face engagement, both during courses and for scholarly communities generally” (ID 33). Almost every respondent mentioned this as the most prominent effect. Adult education itself is clearly in a crisis.

#### Impact on learners, teachers and adult education organisations

Disadvantaged learners were often considered as being even more strongly affected by this lack of provision than others. It was assumed that the reason for this was likely to be that they have less access to digital means of learning and to a supportive social environment of their milieu: Once expert observed that there had been a “[m]ove towards virtual forms of communication, which can be deeply exclusionary by reference to class, age and gender” (ID 42). Gender especially played an important role in some of the comments, such as “Women and carers with childcare responsibilities are additionally discriminated against” (ID 20). Surprisingly, one rare remark from Africa considered this:

“Those who come from depressed socio-economic backgrounds became part of the state-registered vulnerable population. In a sense, COVID-19 served as a social and economic equaliser […]” (ID 7).

The effects of COVID-19 differ between countries and regions, even as the world seems to be united in acknowledging that we have to deal with COVID-19 somehow.

While most comments focused clearly on the effects on *learners,* a number of comments expressed worry about the *organisations, teachers, tutors,* etc.:

- There is an “[u]ncertainty of budgets […] strongly felt in not-for-profit agencies” (ID 2).
- “The cost of the part-time teachers to re-do the work is not likely to be factored into the small adult education budget for 2019/20” (ID 7).
- There is a “serious economic risk for institutions and organisations” (ID 20).
- “Solo self-employed adult educators in many cases fall into basic social security, which does not cover their regular costs” (ID 22).
- There is “economic damage for providers” (ID 37).
- There are “problems with financing” (ID 54).
- “The first effect is cancelling the vast majority of the educational events (lectures, seminars, workshops, etc.). This has serious social effects for adult educators. Many of them are freelancers” (ID 56).

Freelancers and self-employed adult educators were perceived as an especially vulnerable group. Rather surprisingly, however, adult educators with limited or project-based contracts were not mentioned by any expert as vulnerable group. Generally, the situation and development of personnel and organisations of adult education were not given the central role we had expected in the responses.

#### An increase in digital forms of learning

What respondents mentioned almost as frequently as this drop in educational offers and the forced closure of adult education providers was an increase in digital forms of learning (e.g. e-learning, distance learning). The rationality of providers’ decisions is described as in this quote: “Almost all face-to-face learning activities had to be organised at distance, or cancelled” (ID 38). There were a number of observations on extensive further education and training activities for adult educators for digital teaching and digital tutoring. Although the first section of the questionnaire asked about immediate effects, respondents already mentioned likely changes in the near future here: “A new training for teachers, counsellors and other practitioners in adult education and learning will be necessary” (ID 45). Overall, a clear majority of respondents (23 out of 27) rated such an increase of digitalised forms of learning as a very strong effect. Some voices even came to the conclusion or assumption that “[o]nline learning [would be] the new norm” (ID 2) or observed a “boom in the remote learning industry which the adult learning ‘space’ has moved to” (ID 42).

#### Informal learning

Much less frequently mentioned were forms of informal learning or even an increase in informal learning. Nonetheless, some statements related to this were also made:

- “[A]dult learners have a chance to learn from and with their children […] The pandemic forced the families to practise the principle of learning families” (ID 7).
- There is a “[c]urtailment of informal everyday learning from peers” (ID 21).
- “If participation in formal and non-formal learning activities decreases due to dropping of courses and seminars, participation in intentional informal learning increases. (Just estimations, we don´t have any available data yet)” (ID 46).
- The pandemic is “increasing the role of social networks” (ID 48).
- There has been “a ‘silent explosion’ of informal learning (self-help, on-line) oriented to spheres of hobbies and culture” (ID 56).

While informal learning mainly within families and social networks was mentioned by several experts, workplace learning – even via digital tools – was mentioned surprisingly rarely.

#### Changes in the wider context of adult learning and education

Some experts also observed changes in the wider context of adult education or, again, already connected the present with the future in their statements:

- What we are experiencing is “[a] form of cultural shock. If adult education is able to address these issues, it may provide a chance for general political and moral themes in […] adult education” (ID 20).
- What is emerging is “[l]earning as a coping mechanism in isolation” (ID 46).
- “Public investment will be made mainly in the types of adult education that may contribute directly to promoting the economy of the countries […]” (ID 31).
- “The government have promised more money to fund more adult education places as unemployment rates are expected to rise due to COVID” (ID 39).
- “The Ministry of Education and the Ministry of Social Affairs do not have adult education as central priorities of their agenda” (ID 56).
- There is a “[s]hift of the focus of [adult education] content to the health area (viruses, epidemiology, hygiene, immunity, vaccination)” (ID 35).
- There is a “dramatic decrease in critical thinking [due to] the flood of fake news, conspiracy theories, strange behaviours based on non-reflected emotions, etc.” (ID 35).
- “People are more interested in scientific findings and learn intentionally more than before through diverse media channels” (ID 46).

A number of experts also voiced their expectation of *austerity cuts*^8^ by governments in the future. However, one respondent expected or even already referred to their government’s public statement announcing future *increases* in spending for the training of unemployed citizens. By contrast, other governments do seem to have adult education on their agenda.

### Expected effects of COVID-19

In the second part of our questionnaire, we asked the experts about their expectations of the future effects of the COVID-19 crisis on adult education.

#### A further increase of digital learning

A clear majority of experts (22 respondents) expected a further increase towards digital learning:

- “AE [adult education] in companies and large training institutions will continuously shift towards virtual distance teaching” (ID 22).
- A future effect is “[p]robably more telework and virtual learning, because the pandemic crisis proved that other forms of performing professional roles are possible” (ID 31).
- We are likely to see an “[i]ncreased reliance on online educational platforms and contexts” (ID 33).
- We are likely to see an “[i]ncrease in the use of digital tools, and moving learning to the online world, leaving many people behind, especially vulnerable and marginalised groups” (ID 35).
- What is likely in the future is that “[t]here will be more online and distance solutions” (ID 38).
- We are likely to see “[m]ore distance learning as COVID forces all teachers to learn how to teach in distance mode” (ID 39).
- “Providers will offer more digital formats in the future” (ID 46).
- “I am afraid that we will have a flood of online courses and pressure on people to learn online by themselves” (ID 54).
- We are likely to see a “[d]ecrease in a general tendency to be social (and take part in face-to-face educational activities) and it may lead to more alienated training” (ID 56).

With regard to the future, the most frequent assumption seems to be that the COVID-19 crisis is a juncture, introducing major changes or accelerating a general shift towards digital learning. This (new) trend is expected to continue. One respondent expressed the fear that “the main actors are becoming more powerful (Harvard University) and it may lead to an alienated education” (ID 22). Only surprisingly few voices expected a new valorisation of face-to-face learning:

“The importance of face-to-face learning situations (for socialisations, group processes) is realised ‘in a new way’, and it will be valued more, but also thought in more detail” (ID 38).

By contrast, the overwhelming majority of experts did not talk about or did not explicitly mention such a renaissance of or return towards classical forms of adult education with personal encounters in their expectations for the future.

#### Changes in funding priorities

In addition to the switch to online learning, the experts are expecting changes in funding priorities, and increasing demand, especially in the area of health education:

- There is likely to be an “[i]ncrease in importance for the topics digital and healthy [*sic*] literacy (in research and practice)” (ID 14).
- “Moving funds to health education and research” (ID 35) is likely.
- We are likely to see an “[i]ncreasing educational demand for subjects dealing with health education, scientific literacy and crisis awareness” (ID 46).

Only individual respondents mentioned that adult education must do more to promote democracy and human rights, since we have seen how fragile they are (ID 54).

A number of experts expected dire straits for adult education in relation to financing:

- “Uncertainty of resourcing” (ID 2).
- “Less money for education” (ID 16).
- “Less public investment in adult education” (ID 31).
- “Cuts in funding, as government priorities are to housing and food” (ID 34).
- “Cut in funding for various areas of adult education and moving funds to the health education (incl. hygiene), health research, etc.” (ID 35).
- “Publicly provided adult education [is likely] to suffer further as recession bites, and austerity takes firmer hold” (ID 42).
- “I think that financing will decrease even more, but that new self-organised groups will emerge” (ID 54).
- “Adult educators as a group are at risk. Future economic (and social) crises will probably cut the funds and will to invest in education” (ID 56).

These effects were expected in the future by many of our respondents. Compared with the current difficulties, the experts seem to expect even more challenges ahead, mentioning problems with financing slightly more frequently in their responses concerning the future than those concerning immediate effects. Thus, the worst impact in relation to financing adult education might even yet be to come.

#### Increase in inequality

Another development many experts expected in the future was increased inequality:

- There is likely to be “[l]ess access to education for disadvantaged people” (ID 16).
- There is likely to be a “[d]eeper gap between groups of people” (ID 21).
- There is likely to be a “[l]oss of learning opportunities for disadvantaged people” (ID 31).
- “Leaving disadvantaged people behind” (ID 35) is a likely development.
- “Disadvantaged people will be more excluded from the learning possibilities” (ID 55).

Besides raising medical questions and challenges, COVID-19 also confronts us with social and political questions and challenges. The virus does not differentiate between social classes, but the way we deal with the virus and the measures we choose to implement in our attempts to curb infection rates reveals a great deal about our societies and social justice. Experts are very aware of this and they are following core ideas of adult education. Thus, a number of respondents expected an increase in inequalities in the future. This inequality was mainly framed nationally rather than internationally. Increasing differences between nations were mentioned only rarely, though their inclusion might be implicitly assumed when our respondents referred to inequalities generally.

### Summary

The two sets of different survey questions focusing first on observed immediate, present effects and second on expected future effects resulted in astonishingly few differences between responses to both questions. Interestingly, one expert even refused this distinction entirely:

- “Sorry, I answered the first question with a view to future effects. I do not think it makes sense to separate now and [the] future” (ID 20).

Another expert already provided an explanation for the problems connected to this distinction between present and future:

- “Stressing the management of emergence in the present reduces both memory and the imagination of the future, hope and investment on one’s dreams and interests” (ID 16).

Many respondents even seemed to be framed by a kind of *presentism* (Bourne 2006) within this crisis. Presentism is a philosophical concept. It refers to approaches and perceptions which are solely focused on the present without referring to the past or to the future. It seems relevant here, since many experts found it difficult to imagine a future beyond COVID-19 and the present problems. Additionally, others might just not have been able to express it as clearly as those two experts quoted above, since the current problems are of course already substantial and manifold. Overall, then, the differences perceived between the present and future effects were not very pronounced.

The measures put in place to curb the spread of COVID-19 and the many lockdowns and shutdowns of adult education providers’ businesses have touched (and continue to affect) the “heart of adult education”. We found this to be a key quote in our survey, and it is elaborated and reinforced by this statement:

“The adult learning sector – it has been totally challenged and adult learning providers have had to see how they can change their business models, their teaching and learning practices, the way they source for and approach learners, the means with which they engage with learners, etc.“ (ID 51).

Among the responses, there were many reports and statements about the increased importance of distance learning via digital means, although several experts pointed out that this could not totally compensate for closed adult education organisations. Our respondents expect this increase to continue, even in the future. Many warned of the danger that the crisis will likely affect already disadvantaged people even more in the long term. The wider context of adult education is also changing and some experts expressed their concern that authoritarian governments will profit from the crisis and change democratic and legal structures. As observed above, COVID-19 is not only a large-scale medical crisis; it has fundamental social and political implications as well. The funding of adult education is already in danger. This not only applies to the present, but is likely to be exacerbated even more in the future according to a number of responding experts in the first wave of our Delphi study.

While many experts expect that distance learning via digital means will be of increased importance, also in the future, astonishingly, almost no respondent expected a significant renaissance for physical encounters for learning after the crisis. This is to some extent understandable, since it is likely that the crisis will last for many more months or even several years. It is also likely that, for a variety of reasons, such as anxiety, own health problems, living with people at risk, etc., people will for some time be afraid to meet face-to-face again. On the other hand, after months of quarantine and *social distancing*,^9^ there might be a great motivation and eagerness to meet again personally.

Digital solutions introduced during the current pandemic might run into a problem after the crisis if many people become tired of distance education and the technical and social problems connected to it. Clearly – this has become obvious in recent months – while they were crucial in helping to cope with quarantine, digital means also have many pitfalls and limitations (technical, social, financial). Of course, how this pans out in the long term is also dependent on how the crisis and the measures develop over time. If periods of quarantine are introduced again, physical encounters will remain impossible in many respects. There is even the danger that people will become afraid of any physical encounters or that an increase of hypochondriac behaviour will occur. If you follow the thinking of some epidemiologists, meeting people is always dangerous and risky. But life is risky and in the end we will all die. We do not at all mean to underestimate the danger of the pandemic, we are merely pointing out that that *risk competencies*^10^ are also an important issue. We were astonished that many of our respondents currently see no need to build citizens’ skills for informed, critical thinking and critical reading of statistics and for comparing different life risks in order to build their own informed opinion rather than just following medical experts’ and policymakers’ recommendations. Here are two rare examples of comments which did consider the importance of these skills:

- There is an “increase of the need for critical thinking, knowing the background […] At the same time, [there is a] dramatic decrease in critical thinking – the flood of fake news, conspiracy theories” (ID 35).
- “I do not know if this will happen, but we need to advocate even more for adult education for democracy and human rights as we saw how fragile they are” (ID 54).

Nonetheless, many respondents expected an increased interest in health issues even in the future, while other issues (e.g. culture, arts) were rather rarely mentioned. Many experts see a serious danger of a further increase of *vocationalism*^11^ after the crisis. In this context, COVID-19 was quite often constructed as a catalyst or a kind of magnifying glass. The catalyst idea of a sort of forced reaction discerns an acceleration of already existing tendencies like vocationalism or the *digital turn*.^12^ Thus, most respondents also expected an increase of social inequality as a future effect. The idea of a magnifying glass is more visual in that it sees problems which already existed before COVID-19 becoming even more clearly visible during the current pandemic. Poor people die much more frequently from COVID-19 than rich people. And yet, surprisingly, very few respondents suggested that the COVID-19 crisis might lead to social change and, for example, to the introduction of a national basic income as a kind of positive outcome. Nonetheless, it might be crucially important to be aware that a *crisis,* in its original Greek meaning, is a decisive situation, which contains risks but also opportunities.

## Conclusions and outlook

Besides the above-mentioned fluidity between present and future, we also found there were surprisingly few differences between countries, even though we were actually very interested in learning more about these from the experts. Here is another excerpt from the statement already mentioned above from an African respondent:

“COVID-19 served as a social and economic equaliser forcing the state to redistribute the national resources. […] The pandemic has radically changed the way government will contribute to dealing with its adult learners. They are likely to support them with resources even more so that their conditions are improved to avert the spread of the virus if it were to reoccur in future” (ID 7).

This particular expectation deserves more attention, and we have to be very careful to monitor COVID-19 internationally and beyond our national situation and perception. Interestingly, the response rate for our survey from people from large countries such as China and the United States of America (USA) was very low. Thus, there is a serious selection bias within this survey since many voices did not react to the invitation to participate. The next waves of the survey will have to seek new approaches in engaging scholars from China, the USA and other world regions in order to participate in the survey. We have already asked the UNESCO Institute for Lifelong Learning (UIL) in Hamburg for help in this matter and hope for even more support in the future.

Overall, our choice of the Delphi method and the completion of the first wave of the survey have delivered, from our point of view, interesting results. The picture emerging from these results demonstrates the degree and directions of some of the perceived and expected effects. Nonetheless, it has also become clear that the responding experts were also personally surprised by the COVID-19 crisis. They are still trying to understand the present situation and its consequences. It is our impression that the COVID-19 crisis has so far (at the time we are writing this in July 2020) been mainly an issue for medical science, which has delivered medial knowledge and interpretations. By contrast, social sciences and educational science seem to be struggling somewhat in being heard and making sound interpretations. This impression might of course be contestable, but the strong presentism in many of our respondents’ statements does demonstrate that it is currently difficult to imagine what happens next and what will happen in many months’ time.

Using past knowledge does not seem to work very well, even though pandemics are not a totally new occurrence. It is somewhat strange that epidemiologists and economists refer to the “Spanish flu” of 1918 as a key reference point for effective measures (e.g. Garrett 2008; Bootsma and Ferguson 2007), while adult educators do not seem to be able or willing to recall, or to look for, past knowledge which is likely to be relevant to the current situation. Nonetheless, there might be opportunities to learn from the past: How did other people learn and deal with crises? What were the typical mistakes or successes in the past? Is the present crisis really that unique? Is dealing with crises not somehow a key characteristic of adult education, which very often has to deal with fundamental transitions and transformations of individuals, collectives or societies? Are all measures against COVID-19 really reasonable and effective? What is the “collateral damage” of the measures? What will this mean for the potential next waves of the pandemic? Does adult education provide a forum for thinking and debating measures and alternatives? Do we not need interdisciplinary approaches? Or do we confine ourselves to trusting in epidemiologists and policymakers? Again, there is a tendency of presentism observable within the data we collected in the first wave of our Delphi study.

There are many dangers and some opportunities. We contacted the experts in a situation while they themselves were at the limits of their knowledge and struggling to make sense of the developments and changes. Relying on past experiences might no longer work sufficiently in many respects or might even be substantially misleading, although this is debatable, as already mentioned. Thus, compared to “normal” situations, experts cannot draw that much on knowledge, but rather need to rely on observations and educated guesses. This is definitely not a critique of the experts, but it underlines how important it is to stimulate and maintain exchange within the scientific community even while conferences have been cancelled and many activities have been postponed. To some extent, the (social) sciences seem to be paralysed under the shock of COVID-19. With this Delphi study, we wish to contribute to processing this shock and to support international dialogue and exchanges through the implementation and completion of the next waves of our Delphi study. At the end of our questionnaire (see methods section), we asked people for any other comments or recommendations, also for our construction of the questions for the next two waves of our Delphi study, and for their advice on what we or adult education in general should do in the future:

- “A clearly pressing question is how our awareness of socially and medically vulnerable groups can be enhanced and maintained. I think that is an AE project we could all get our teeth into!” (ID 2).
- One respondent advised that we should “have exploratory questions on how state and private funding of adult learning could be” (ID 7).
- “For instance, I would have mentioned a campaign in North America to sign a document entitled ‘Human mobility and human rights in the COVID-19 pandemic: Principles of protection for migrants, refugees, and other displaced persons’” (ID 14).
- “I would try to chronicle the different areas in more specific ways, asking about access, changes due to the massive use of e-learning, new organisational challenges, the social and psychological effects of the reduction of social contacts” (ID 16).
- “It would be reasonable to focus attention on the ‘apocalyptic’ nature of this crisis” (ID 20).
- One respondent suggested including this question: “What issues should be prioritised in adult education research agenda in the near future due to the COVID-19 pandemic situation?” (ID 21).
- Another respondent’s advice was to “[f]ocus on the risks that the COVID-19 pandemic brought to adult learning, especially how it will be used by authoritarian governments in many countries, and what adult education could do about it!” (ID 35).
- “Think big, but also realistically. Adult education is finding new forms of expression – although not necessarily under that name – which should be chronicled and theorised. But it will continue to struggle in a formal sense, as the world plunges into recession” (ID 42).
- One respondent suggested including this question: “Is there any way countries could come together to pool resources in order to better steer adult education and adult learning towards the best outcomes possible?” (ID 51).
- “Including questions like how adult education helped overcome or minimise the effect of COVID-19 may help us understand the positive contributions” (ID 53).
- “I think the more meaningful question is to ask adult educators about what should be the emphasis of adult learning and adult education after the outbreak” (ID 54).
- Finally, one expert pointed out that “[t]he tendencies you explore can be different in the fields of a) practice of education and learning, b) policy and politics and c) thinking of people” (ID 56).

Thus, the experts contributed a wide array of interesting and encouraging ideas. As we write this, we are wondering which of these ideas expressed by our respondents will most strike the reviewers and readers of our article? It will be a pleasure – but also a challenge – to decide how many of these outlined routes can be followed.

In sum, our findings demonstrate that COVID-19 is currently having fundamental effects which will continue to impact on future adult education and adult learning. A forced digitalisation, more inequality and greater financial constraints are some of the core issues mentioned by many of our respondents. The experts themselves were at times clearly struggling to understand the situation. They rarely used past knowledge and crises as a background for their reflections on their experiences, since the current situation is often framed as being unique. COVID-19 is at times seen as something between a catalyst and a magnifying glass. The ongoing COVID-19 pandemic is further increasing already existing social and political problems. This makes its impact even worse than its characteristic of being “only” a medical crisis.

Additionally, it is important to note that during the implementation of the first wave of our Delphi study, and while we are writing the article, the situation has already changed and is still changing. While during the early phase of the pandemic, the main affected regions were the Northern hemisphere with China and especially Europe, it is now (in July 2020) rather the Southern hemisphere and the USA which are affected most. While the whole world is affected by COVID-19, infection rates vary and often occur asynchronously. These developments provide all the more reason to continue research on the worldwide effects of COVID-19 with a survey method like a Delphi study which, with its different waves of questionnaires and mutual expert feedback loops, is particularly well suited to capture a constantly evolving crisis over time.

## Footnotes

1 The term “forced digitalisation” refers to the acceleration effect of the current conditions on often still rather nascent digitalisation strategies and policies in companies, institutions and at government level, who are now galvanised – or “forced” – into action and much more elaboration.

2 The exact wording of this is not known (QI 2013), but the gist of it is established and we quote the most commonly used version here to make our point.

3 Quoted by Stansberry (2011), without providing a source.

4 Scheduled for 4–7 June 2020 at the University of British Columbia in Vancouver, Canada, AEGT2020 itself had to be cancelled on 17 March due to the pandemic. However, all presentations which had already been accepted for the conference were published online in a 780-page *Proceedings* volume (Egan 2020).

5 We are unable to provide more information here since respondents were assured of our confidentiality.

6 This article was drafted in July 2020.

7 To maintain confidentiality, we reference our respondents’ statements by ID, discerning them by numbers.

8 An austerity cut is a reduction in government spending, in some cases coupled with increases in tax revenues. Especially public organisations and social services are often strongly affected by such cuts and have to reduce their offers or have to raise fees for participants.

9 Somewhat misnamed, what “social distancing” actually means is keeping a *physical* distance from other human beings to reduce transmission of the COVID-19 virus. In the current pandemic, the stipulated space varies across countries; a fairly common distance is 1.5 to 2 metres. However, the effect of adhering to physical distancing directives has indeed also been social isolation, especially of vulnerable and elderly citizens.

10 The term “risk competencies” refers to understanding and assessing risks in a wider context, or to the ability of gauging different risks in a skilled way during decision-making (Gigerenzer 2015).

11 Vocationalism refers to an agenda prioritising such forms of adult education and adult learning which seem to be directly applicable in the world of work and are thus regarded as being “useful” and utilitarian. Liberal education and critical thinking are somewhat neglected by this approach and underestimated both in terms of their own value and in terms of the interwovenness of general and vocational knowledge and skills.

12 In the context of (adult) education, he term “digital turn” not only refers to the replacement of many learning tools, such as paper, pencils and books, with electronic devices, but also to the transformation of learning processes resulting from this shift, as well as both new opportunities and new problems and limits which arise (Hofhuis 2018).
